# Diagnostic Performance of Color Doppler Ultrasound for Detecting Nuchal Cord and Associated Immediate Neonatal Outcomes at Term in Uganda: A Prospective Diagnostic‐Accuracy Cohort Study

**DOI:** 10.1002/hsr2.73385

**Published:** 2026-10-13

**Authors:** Fiona Asiimwe, Theoneste Hakizimana, Marie Pascaline Sabine Ishimwe, Kato Paul Kalyebara, Leevan Tibaijuka, Joseph Ngonzi, Henry Lugobe, Godfrey Twesigomwe, Kahimakazi Irene, Jjuuko Mark, Musa Kayondo, Julius Mugisha, Rogers Kajabwangu

**Affiliations:** ^1^ Department of Obstetrics and Gynaecology Mbarara University of Science and Technology Mbarara Uganda; ^2^ Department of Obstetrics and Gynaecology Kampala International University Bushenyi Uganda; ^3^ Department of Pediatrics and Child Health Kampala International University Bushenyi Uganda

**Keywords:** color Doppler ultrasonography, diagnostic performance, intrapartum ultrasound, neonatal outcomes, Nuchal cord, term pregnancy, Uganda

## Abstract

**Background and Aims:**

Evidence on the diagnostic performance of color Doppler ultrasound for detecting nuchal cord and its association with immediate neonatal outcomes remains limited in sub‐Saharan Africa. We evaluated ultrasound detection of nuchal cord and compared immediate neonatal outcomes according to nuchal‐cord status.

**Methods:**

This prospective study included 246 women with singleton pregnancies at ≥ 37 weeks who were enrolled in early labor at Mbarara Regional Referral Hospital, Uganda, from February to July 2021. Two‐dimensional and color Doppler ultrasound was performed at enrollment, and direct examination of the umbilical cord at delivery was the reference standard. Diagnostic‐performance measures and unadjusted risk ratios (RRs) were reported with 95% confidence intervals (CIs).

**Results:**

Nuchal cord was confirmed at delivery in 88/246 participants in the sampled cohort. Ultrasound identified 76/88 participants with and 152/158 without a nuchal cord. Sensitivity was 86.4% (95% CI: 77.4–92.8), specificity 96.2% (95% CI: 91.9–98.6), positive predictive value 92.7% (95% CI: 84.8–97.3) and negative predictive value 92.7% (95% CI: 87.6–96.2). Overall accuracy was 92.7% (228/246), and the area under the receiver operating characteristic curve was 0.913. The composite immediate adverse neonatal outcome occurred in 18/88 neonates with a nuchal cord and 8/158 without one (20.5% vs. 5.1%; RR: 4.04, 95% CI: 1.83–8.91).

**Conclusion:**

Color Doppler ultrasound demonstrated good diagnostic performance for detecting nuchal cord at term. Nuchal cord was associated with higher unadjusted risks of immediate adverse neonatal outcomes, although residual confounding cannot be excluded. Sonographic detection may inform delivery‐room preparedness but should not, in isolation, determine the mode of delivery.

## 1 Introduction

A nuchal cord occurs when the umbilical cord encircles the fetal neck by at least 360° and may involve one or multiple loops [1, 2]. It is a common obstetric finding whose frequency increases with advancing gestation because of fetal movement, cord length, and changing fetoplacental spatial relationships [3, 4]. Reported prevalence varies according to gestational age, population characteristics, timing and method of assessment, but nuchal cord is identified in approximately 10%–35% of term deliveries [1, 5, 6]. Although most isolated nuchal cords do not result in clinically important fetal compromise, their antenatal detection frequently raises concern among pregnant women and clinicians because of the perceived risk of cord compression, fetal hypoxia and adverse neonatal outcomes [7, 8].

A nuchal cord can be detected antenatally using gray‐scale ultrasonography, with color Doppler imaging used to confirm blood flow within the umbilical‐cord vessels surrounding the fetal neck. Direct examination of the cord at delivery remains the clinical reference standard. Diagnostic performance is conventionally described using sensitivity, specificity, positive and negative predictive values and overall accuracy, together with confidence intervals (CIs) to communicate the precision of these estimates [9]. Color Doppler generally performs better than gray‐scale imaging alone because it improves visualization of the cord vessels and their relationship to the fetal neck. Jauniaux et al. [6] reported that sensitivity increased from 33% with gray‐scale imaging to 79% with color Doppler and was higher after 36 weeks of gestation. Other prospective studies have reported high sensitivity, specificity and negative predictive values for color Doppler at term or during labor [10, 11, 12].

Nevertheless, the diagnostic performance reported across studies has varied substantially. Peregrine et al. [13], for example, reported a sensitivity of only 37.5% immediately before induction of labor, despite a specificity of 80%. Differences in gestational age, fetal head position, number of cord loops, ultrasound technique, operator experience and the interval between ultrasound assessment and delivery may contribute to this variation. Furthermore, nuchal cords are dynamic: they may form, resolve or change in the number of loops between examination and delivery [3, 6]. Diagnostic‐accuracy estimates derived from one population or examination protocol may therefore not be directly generalizable to other clinical settings.

The potential clinical role of ultrasound during labor extends beyond detecting a nuchal cord. Intrapartum ultrasound can provide objective information on fetal head position, station, attitude, descent and rotation and may assist in evaluating labor progress and planning operative vaginal delivery [14]. Ultrasonography combined with color or power Doppler is also increasingly used to assess other umbilical‐cord abnormalities, including true knots, abnormal cord insertion, cord presentation and complex cord entanglement [2, 15]. However, ultrasound cannot reliably visualize the entire umbilical cord in every pregnancy. A sonographic nuchal‐cord finding should therefore be interpreted alongside fetal surveillance and labor progress rather than used as an isolated indication for induction or operative delivery.

The association between nuchal cord and adverse neonatal outcomes remains uncertain. During fetal descent, stretching of a tightly encircling cord may compress the umbilical vessels, potentially causing transient fetal heart‐rate abnormalities, reduced fetal oxygenation or impaired neonatal adaptation [1, 16]. Some studies have reported increased neonatal resuscitation, low Apgar scores or neonatal‐unit admission among neonates with a nuchal cord, particularly when the cord is tight or involves multiple loops [4, 5]. Conversely, other studies have found no clinically meaningful differences in mode of delivery or immediate neonatal outcomes between pregnancies with and without an isolated nuchal cord [7, 13]. These inconsistent findings suggest that the presence of a nuchal cord alone may be less clinically informative than its tightness, number of loops, associated fetal heart‐rate abnormalities and the availability of timely intrapartum and neonatal care.

Evidence on the diagnostic performance of color Doppler ultrasound for detecting nuchal cord remains limited in Uganda and similar resource‐constrained settings. Differences in equipment, operator expertise, fetal‐monitoring capacity and access to emergency obstetric and neonatal care may affect both diagnostic performance and clinical outcomes. Locally generated evidence is therefore needed to support appropriate maternal counseling, delivery‐room preparedness and avoidance of unnecessary obstetric intervention. This study aimed to determine the diagnostic accuracy of two‐dimensional and color Doppler ultrasound for detecting nuchal cord among women with singleton term pregnancies admitted in early labor at Mbarara Regional Referral Hospital (MRRH), using direct examination at delivery as the reference standard. The study also explored associations between nuchal‐cord status at delivery and immediate neonatal outcomes.

## 2 Materials and Methods

### 2.1 Study Design and Setting

This prospective cohort study was conducted from February 2021 to July 2021 at MRRH, a public tertiary and teaching hospital for Mbarara University of Science and Technology, located 260 km southwest of Kampala. MRRH serves over 5 million people across 12 districts and neighboring countries (Rwanda, Burundi, Tanzania, DRC) and conducts approximately 12,000 deliveries annually.

The Department of Obstetrics and Gynecology provides 24‐h intrapartum care with 13 obstetricians, 31 residents, 10 interns, and 16 midwives. The labor suite and theatres are equipped with ultrasound (2D and color Doppler), neonatal resuscitation stations (ambu‐bags, suction bulbs, warmers, oxygen), and Apgar score charts. A neonatal unit, located within 20–80 m, enables rapid transfer of newborns requiring specialized care.

### 2.2 Study Population

The study included consenting pregnant women at ≥ 37 weeks of gestation, determined from the first day of the last normal menstrual period or first‐trimester ultrasound, who were admitted in early labor with a live singleton fetus. Restriction to term singleton pregnancies reduced heterogeneity associated with prematurity and multiple gestation, while enrollment in early labor permitted standardized ultrasound assessment before advanced fetal descent and prospective follow‐up until delivery.

Women were excluded if they had multiple gestations, confirmed intrauterine fetal death, a pre‐existing indication for caesarean delivery, or umbilical‐cord entanglement involving multiple fetal body parts, such as the neck and trunk or neck and limbs. These conditions were excluded because they could complicate sonographic classification, independently influence neonatal outcomes, preclude assessment of liveborn neonatal outcomes, or determine the mode of delivery independently of the ultrasound finding.

### 2.3 Sample Size Determination

Sample size was estimated for both objectives, and the larger estimate was adopted. For the primary diagnostic‐accuracy objective, the Buderer method was applied assuming 95% sensitivity, 90% specificity, 34% nuchal‐cord prevalence, 5% absolute precision and a 95% confidence level [12, 17]. The larger, sensitivity‐based estimate was 215 participants; adjustment for 10% potential nonresponse or incomplete data increased this to 239.

For the secondary objective, the cohort sample‐size procedure in OpenEpi version 3.01 was used. Neonatal intensive care unit admission was expected in 17% of neonates with a nuchal cord and 5% of those without, based on Meena et al [18]. At 80% power, a two‐sided 95% confidence level and a 1:2 group ratio, 222 participants were required. Allowing 10% attrition and maintaining this ratio produced a final target of 246 participants: 82 with and 164 without a nuchal cord. This larger estimate was adopted.

### 2.4 Diagnosis of Nuchal Cord

The index test was two‐dimensional ultrasound combined with color Doppler imaging. All examinations were performed using the same GE LOGIQ e portable ultrasound machine (GE Medical Systems, China), equipped with a 3.5‐MHz 4C‐RS convex transducer and standardized preset settings. Participants were examined in a semi‐recumbent position. The fetal neck was assessed in transverse and sagittal planes, initially using gray‐scale imaging to identify the “divot sign.” Color Doppler was then applied using a low wall filter, gain adjusted to just below background noise and an appropriately scaled pulse‐repetition frequency to demonstrate arterial and venous flow within the umbilical‐cord vessels.

A positive finding was defined as visualization of umbilical‐cord vessels along at least three of the four quadrants of the fetal neck, with color Doppler confirmation of blood flow within the vessels. Examinations that did not meet these criteria were classified as negative. The number of cord loops identified was documented. Unclear findings were independently reviewed by a second radiologist before final classification.

The reference standard was direct examination of the fetal neck and umbilical cord at delivery. Delivery staff documented the presence or absence of a nuchal cord, together with the number and tightness of any loops, before accessing the ultrasound findings.

### 2.5 Sampling Technique

Eligible women admitted in early labor (cervical dilatation < 6 cm) were screened consecutively and enrolled after providing informed consent. Recruitment used a predetermined 1:2 ratio based on the index‐test result: for each participant with an ultrasound‐positive finding for nuchal cord, two consecutive participants with an ultrasound‐negative finding were enrolled. Recruitment continued until the target sample of 246 participants was achieved, comprising 82 ultrasound‐positive and 164 ultrasound‐negative participants. Because enrollment was stratified by the ultrasound result, selection was not independent of the index test. All enrolled participants underwent two‐hourly clinical reviews until delivery, and delivery and immediate neonatal outcomes were recorded.

### 2.6 Data Collection Procedure

On admission, potentially eligible women underwent focused history taking, obstetric examination, and vaginal examination. After written informed consent was obtained, sociodemographic and obstetric information was collected using a coded interviewer‐administered questionnaire. Each participant underwent one standardized two‐dimensional and color Doppler ultrasound examination at enrollment in early labor, defined as cervical dilatation < 6 cm. The examination procedure and diagnostic criteria are described under Diagnosis of Nuchal Cord. Participants were subsequently followed clinically at 2‐h intervals until delivery. These assessments formed part of routine labor monitoring and did not include repeated research ultrasound examinations. Fetal heart rate, cervical dilatation, membrane and liquor status, and intrapartum interventions were documented. The ultrasound procedure and follow‐up requirements were explained during the consent process, and participation did not delay, replace or interfere with routine obstetric care.

At delivery, trained staff recorded the mode and time of birth and directly examined the fetal neck and umbilical cord for the presence, number and tightness of nuchal‐cord loops. Delivery staff remained blinded to the ultrasound findings until these observations had been documented. Neonatal data included sex, birth weight, Apgar scores at 1 and 5 min, neonatal resuscitation, neonatal‐unit admission within 1 h and intrapartum stillbirth after enrollment. Participants were followed until 1 h after delivery to ascertain the prespecified immediate neonatal outcomes.

### 2.7 Study Variables

For the primary diagnostic‐performance analysis, the target condition was the presence of at least one complete loop of the umbilical cord around the fetal neck, confirmed by direct examination at delivery. The index test was two‐dimensional ultrasound combined with color Doppler imaging.

For the secondary exploratory analyses, the exposure was the presence of a nuchal cord at delivery; neonates without a nuchal cord constituted the reference group. The prespecified immediate neonatal outcomes were neonatal resuscitation after birth, a 5‐min Apgar score < 7, neonatal‐unit admission within 1 h and intrapartum stillbirth after enrollment. The composite immediate adverse neonatal outcome was defined as the occurrence of at least one of these outcomes.

### 2.8 Data Quality Control

Before data collection, research assistants who were medical doctors received training in participant screening, informed consent, standardized data collection and study‐outcome definitions. Ultrasound examinations were performed by a radiologist using the same GE LOGIQ e machine, standardized preset settings and predefined acquisition and interpretation criteria. Unclear findings and a random 10% sample of stored images were independently reviewed by a second radiologist who was blinded to the delivery findings. Interpretive disagreements were reviewed by a gynecologist in consultation with both radiologists, and the final classification was reached by consensus. This process provided qualitative quality assurance; a formal interobserver agreement coefficient was not calculated. Intraobserver reliability was not assessed because examinations were not routinely repeated and interpreted by the same radiologist.

Delivery staff remained blinded to the ultrasound results until they had documented the presence or absence of a nuchal cord, the number of loops and whether the cord was tight or loose. Immediate neonatal outcomes, including Apgar scores, neonatal resuscitation and neonatal‐unit admission, were recorded on standardized forms by two independent observers. Discrepancies were checked against the clinical records and resolved by the principal investigator. Completed questionnaires and study forms were reviewed daily for completeness and consistency and cross‐verified against partographs, labor‐ward records and delivery registers. All 246 participants had complete ultrasound, delivery and immediate neonatal‐outcome data and were included in the final analysis.

### 2.9 Data Management and Analysis Plan

Completed questionnaires were checked daily for completeness and stored in a locked cabinet with restricted access. Data were double‐entered into REDCap, compared with source documents and cleaned using range, consistency and logic checks before exportation to Stata version 14 (StataCorp, College Station, TX, USA). All 246 enrolled participants had complete index‐test, reference‐standard and immediate neonatal‐outcome data.

Approximately normally distributed continuous variables were summarized using means and standard deviations, whereas skewed continuous variables were summarized using medians and interquartile ranges. Categorical variables were summarized using frequencies and percentages. Continuous variables were compared using the independent‐samples Student's *t* test when approximately normally distributed. Categorical variables were compared using Pearson's Chi‐square test or Fisher's exact test when expected cell counts were small. The prespecified primary analysis evaluated the diagnostic performance of two‐dimensional and color Doppler ultrasound for detecting nuchal cord, using direct examination at delivery as the reference standard. Sensitivity, specificity, positive predictive value, negative predictive value and overall accuracy were calculated from a 2 × 2 contingency table and reported with 95% CIs. Overall accuracy was defined as the proportion of participants correctly classified. The AUC was reported separately.

Comparisons of immediate neonatal outcomes according to nuchal‐cord status were secondary exploratory analyses. Unadjusted RRs with 95% CIs were reported. Fisher's exact test was used when expected cell counts were small. All statistical tests were two‐sided, and *p* < 0.05 was considered statistically significant. Findings were interpreted primarily according to the magnitude and precision of effect estimates, with *p* values treated as supplementary measures of statistical evidence.

### 2.10 Ethical Considerations

Ethical approval was obtained from the Mbarara University of Science and Technology Research Ethics Committee on 14 January 2021 (MUST‐2021‐005). Administrative permission was obtained from MRRH before study commencement. Written informed consent was obtained from each participant before enrollment. Participation was voluntary, and declining or withdrawing from the study did not affect the care received. Participant confidentiality was protected using unique study‐identification codes, restricted access to study records and de‐identified electronic data. The study was conducted in accordance with the Declaration of Helsinki and the protocol approved by the Research Ethics Committee.

## 3 Results

Of the 300 women assessed for eligibility, 54 were excluded: 24/300 (8.0%) had confirmed intrauterine fetal death and 30/300 (10.0%) had breech presentation. The remaining 246 women were enrolled in early labor and included in the analysis (Figure 1). At delivery, nuchal cord was confirmed in 88/246 neonates (35.8%) in the sampled cohort, while 158/246 (64.2%) had no nuchal cord.

**Figure 1:**
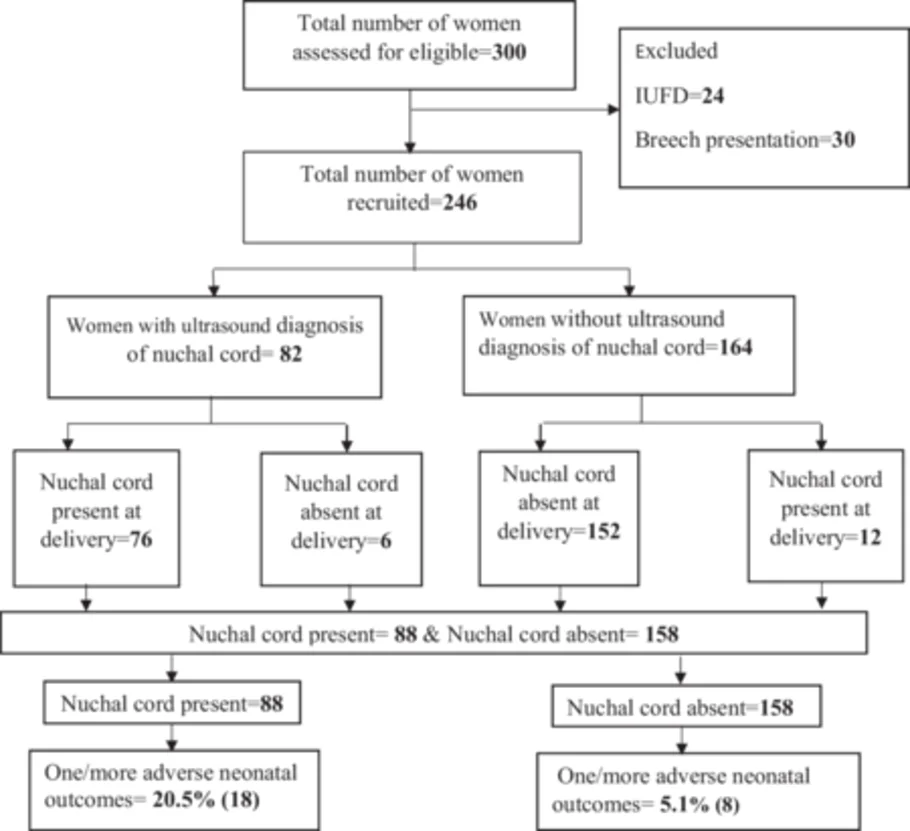
Participant flow through the prospective diagnostic‐accuracy cohort study. Of the 300 women assessed for eligibility, 24 were excluded because of confirmed intrauterine fetal death and 30 because of breech presentation. The final analysis included 246 women enrolled in early labor: 82 with an ultrasound‐positive finding and 164 with an ultrasound‐negative finding for nuchal cord. All enrolled participants completed the index test, reference‐standard assessment at delivery and immediate neonatal‐outcome assessment.

Primigravidity was more frequent among women whose neonates had a nuchal cord than among those whose neonates did not (47/88 [53.4%] vs. 63/158 [39.9%]; *p* = 0.04). Mode of delivery also differed between groups. Caesarean delivery occurred in 33/88 women in the nuchal‐cord group (37.5%) and 15/158 in the no‐nuchal‐cord group (9.5%), while vacuum extraction occurred in 3/88 (3.4%) and 2/158 (1.3%), respectively. Spontaneous vaginal delivery occurred in 52/88 women in the nuchal‐cord group (59.1%) and 141/158 in the no‐nuchal‐cord group (89.2%; overall *p* < 0.001; Table 1).

**Table 1:** Maternal and obstetric characteristics according to nuchal‐cord status at delivery.

Characteristic	Nuchal cord (*N* = 88) *n*/*N* (%)	No nuchal cord (*N* = 158) *n*/*N* (%)	*p*‐value
Age in years, mean (±SD)	25.72 ± 6.27	25.46 ± 5.91	0.955
Gravidity, *n* (%)			0.041*
Primigravida	47 (53.4)	63 (39.9)	
Multigravida	41 (46.6)	95 (60.1)	
Gestational age, *n* (%)			0.822
37–40 weeks	72 (81.8)	132 (83.5)	
> 40–42 weeks	14 (15.9)	24 (15.2)	
> 42 weeks	2 (2.3)	2 (1.3)	
Delivery mode, *n* (%)			< 0.001*
SVD	52 (59.1)	141 (89.2)	
Cesarean section	33 (37.5)	15 (9.5)	
Vacuum extraction	3 (3.4)	2 (1.3)	

*Note:* Data are presented as mean ± SD or *n*/N (%). The independent‐samples Student's *t* test was used for maternal age. Pearson's Chi‐square test was used for gravidity and mode of delivery, and an exact test was used for gestational‐age categories because of small expected cell counts. All tests were two‐sided.

Abbreviations: SD, standard deviation; SVD, spontaneous vertex delivery.

Using direct examination at delivery as the reference standard, ultrasound correctly classified 228/246 participants, giving an overall accuracy of 92.7%. Sensitivity was 86.4% (76/88; 95% CI: 77.4–92.8), specificity was 96.2% (152/158; 95% CI: 91.9–98.6), positive predictive value was 92.7% (76/82; 95% CI: 84.8–97.3), and negative predictive value was 92.7% (152/164; 95% CI: 87.6–96.2; Tables 2 and 3). The area under the receiver operating characteristic curve was 0.913 (Figure 2).

**Table 2:** Cross‐classification of ultrasound findings and nuchal‐cord status at delivery.

		Direct examination at delivery (reference standard)		
		**Nuchal cord present**	**Nuchal cord absent**	**Total**
Ultrasound (index test)	Nuchal cord present	76 (True positive)	6 (False positive)	82
	Nuchal cord absent	12 (False negative)	152 (True negative)	164
	Total	88	158	246

*Note:* The index test was two‐dimensional ultrasound combined with color Doppler imaging. Direct examination of the fetal neck and umbilical cord at delivery was the reference standard. Values are numbers of participants.

**Table 3:** Diagnostic performance of two‐dimensional and color Doppler ultrasound for detecting nuchal cord.

Ultrasound	*n*/*N* (%)	%	95% CI
Sensitivity	76/88	86.4	77.4–92.8
Specificity	152/158	96.2	91.9–98.6
Positive predictive value	76/82	92.7	84.8–97.3
Negative predictive value	152/164	92.7	87.6–96.2

**Figure 2:**
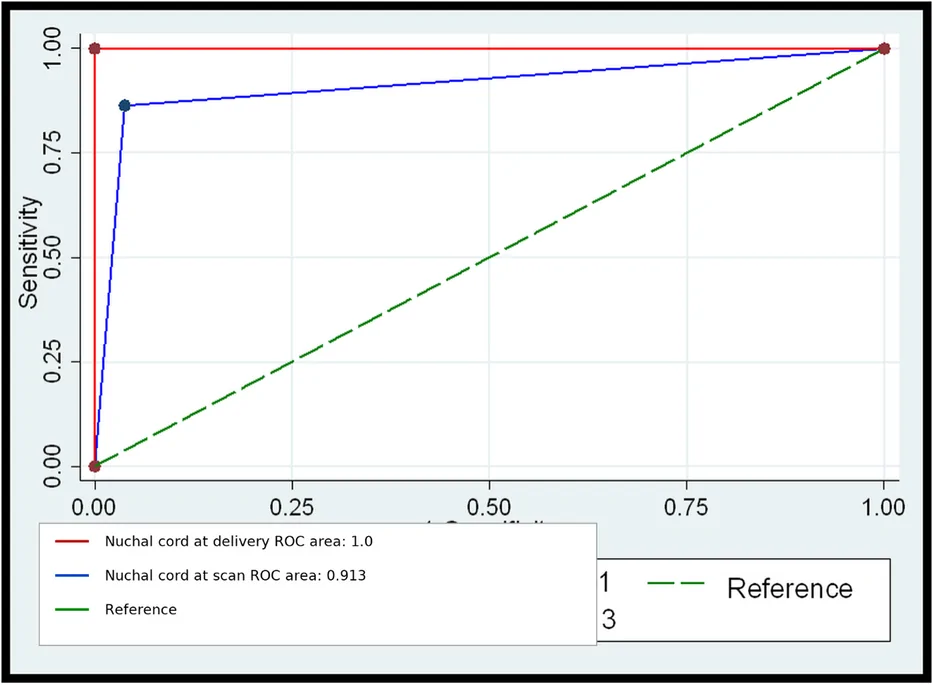
Receiver operating characteristic curve for color Doppler ultrasound detection of nuchal cord. The curve summarizes the ability of two‐dimensional and color Doppler ultrasound, used as the index test, to distinguish participants with and without a nuchal cord confirmed by direct examination at delivery. The area under the receiver operating characteristic curve was 0.913. AUC, area under the curve; ROC, receiver operating characteristic.

The composite immediate adverse neonatal outcome defined as neonatal resuscitation, 5‐min Apgar score < 7, neonatal‐unit admission within 1 h or stillbirth occurred in 18/88 neonates with a nuchal cord (20.5%) and 8/158 without a nuchal cord (5.1%; unadjusted RR: 4.04, 95% CI: 1.83–8.91; *p* < 0.001). Neonatal resuscitation was required for 17/88 and 8/158 neonates, respectively (19.3% vs. 5.1%; unadjusted RR: 3.82, 95% CI: 1.72–8.48; *p* < 0.001). Neonatal‐unit admission occurred in 13/88 neonates with a nuchal cord (14.8%) and 5/158 without a nuchal cord (3.2%; unadjusted RR 4.67, 95% CI: 1.72–12.66; *p* = 0.001). A 5‐min Apgar score < 7 occurred in 1/88 and 1/158 neonates, respectively (1.1% vs. 0.6%; unadjusted RR: 1.80, 95% CI: 0.11–28.35; two‐sided Fisher's exact *p* > 0.99). The wide CI reflected substantial uncertainty because events were rare. No stillbirths occurred in either group (Table 4).

**Table 4:** Comparison of the difference in the proportion of adverse neonatal outcomes between women with and without a diagnosis of nuchal cord.

Outcome	Nuchal cord, *n*/*N* (%)	No nuchal cord, *n*/*N* (%)	Unadjusted RR (95% CI)	*p*‐value
Composite immediate adverse neonatal outcome	18/88 (20.5)	8/158 (5.1)	4.04 (1.83–8.91)	*p* < 0.001
Neonatal resuscitation	17/88 (19.3)	8/158 (5.1)	3.82 (1.72–8.48)	*p* < 0.001
Neonatal‐unit admission within 1 h	13/88 (14.8)	5/158 (3.2)	4.67 (1.72–12.66)	*p* = 0.001
5‐min Apgar score < 7	1/88 (1.1)	1/158 (0.6)	1.80 (0.11–28.35)	*p* > 0.99
Intrapartum stillbirth after enrollment	0/88 (0.0)	0/158 (0.0)	Not estimable	—

*Note:* The composite outcome was defined as the occurrence of at least one of the following: neonatal resuscitation within 5 min, 5‐min Apgar score < 7, neonatal‐unit admission within 1 h or intrapartum stillbirth after enrollment. RRs are unadjusted. Fisher's exact test was used for the 5‐min Apgar comparison. An RR could not be estimated for intrapartum stillbirth because no events occurred.

Abbreviations: CI, confidence interval; RR, risk ratio.

Figure 1 represents the study flow chart. Table 1 summarizes baseline characteristics by nuchal‐cord status. Tables 2 and 3 presents diagnostic performance metrics. Table 4 shows neonatal outcomes.

## 4 Discussion

This study had three principal findings. First, two‐dimensional ultrasound combined with color Doppler correctly classified 228 of 246 participants, corresponding to an overall accuracy of 92.7%, with 86.4% sensitivity and 96.2% specificity. Second, the composite immediate adverse neonatal outcome occurred more frequently among neonates with a nuchal cord than among those without one. Third, this difference was driven mainly by greater requirements for neonatal resuscitation and neonatal‐unit admission, whereas a 5‐min Apgar score below 7 was rare and no stillbirths occurred after enrollment. Because the neonatal‐outcome comparisons were secondary exploratory analyses based on unadjusted risk ratios, they should be interpreted as associations rather than evidence that nuchal cord directly caused these outcomes.

The sensitivity of 86.4% indicates that ultrasound identified 76 of the 88 nuchal cords confirmed at delivery, while 12 were missed. The specificity of 96.2% indicates that 152 of the 158 neonates without a nuchal cord were correctly classified, with six false‐positive findings. These results are broadly consistent with previous evidence showing improved detection when color Doppler is added to gray‐scale imaging. Aksoy reported sensitivity of 95% and specificity of 92% [10], while Jauniaux et al. [6] found that color Doppler was more sensitive than gray‐scale imaging and that detection improved after 36 weeks of gestation. Qin et al. [12] also reported high diagnostic performance when color Doppler was used during labor [12]. The present sensitivity was lower than that reported by Ibrahim et al. [11] but substantially higher than the 37.5% reported by Peregrine et al. [13] immediately before induction of labor. Differences in gestational age, fetal head position, number of loops, operator experience, scanning technique and the interval between ultrasound assessment and delivery may account for this variability. Nuchal cords may also form, resolve or change in loop number during this interval [3, 6].

The positive and negative predictive values were both 92.7% in the sampled cohort. However, predictive values depend on the frequency of the condition and may differ in populations with different nuchal‐cord distributions. Similarly, the area under the receiver operating characteristic curve was 0.913, whereas the proportion correctly classified was 92.7%; these are related but distinct measures and should be reported separately. Because participants were recruited using a predetermined ratio based on the ultrasound finding, the observed proportion with a nuchal cord should not be interpreted as population prevalence. This sampling approach may also have influenced the diagnostic‐performance estimates; validation in a fully consecutive cohort recruited independently of the index‐test result is therefore required.

Intrapartum ultrasound has applications beyond detecting nuchal cord. It can provide an objective assessment of fetal head position, station, attitude and descent and may support clinical decision‐making when labor progress is uncertain or operative vaginal delivery is being considered [14]. Gray‐scale, color and power Doppler imaging are also increasingly used to assess other umbilical‐cord abnormalities, including abnormal cord insertion, vessel‐number abnormalities, cord presentation, true knots and complex cord entanglement [2, 15]. Nevertheless, the entire umbilical cord cannot always be visualized, and diagnostic performance depends on the abnormality, examination timing and operator expertise. Sonographic findings should therefore be interpreted alongside labor progress and fetal surveillance rather than used in isolation to determine the timing or mode of delivery.

The composite immediate adverse neonatal outcome occurred in 18 of 88 neonates with a nuchal cord and 8 of 158 without one. This corresponded to an approximately fourfold higher unadjusted risk among neonates with a nuchal cord. A biologically plausible explanation is that stretching or tightening of the cord during fetal descent may compress the umbilical vessels, reduce fetal blood flow and produce hypoxia, hypercapnia, or fetal‐heart‐rate abnormalities that impair immediate neonatal adaptation [1, 16]. The risk may be greater with tight or multiple loops than with an isolated loose loop. However, the present estimates were unadjusted, and potential influences such as Primigravidity, mode of delivery, loop number, cord tightness, labor duration and intrapartum fetal‐heart‐rate abnormalities were not accounted for. Residual confounding may therefore explain part of the observed association.

Neonatal resuscitation was required in 19.3% of neonates with a nuchal cord and 5.1% of those without one, while neonatal‐unit admission occurred in 14.8% and 3.2%, respectively. Mariya et al. [19] similarly reported that multiple‐part umbilical‐cord entanglement was associated with increased neonatal resuscitation requirements. These findings support preparedness for immediate neonatal assessment and resuscitation when a nuchal cord is suspected. Nevertheless, resuscitation and neonatal‐unit admission are related outcomes and may reflect institutional practice, staffing, admission criteria and clinician judgment in addition to neonatal condition. Neonatal‐unit admission should consequently be interpreted as an indicator of additional care rather than an independent biological complication of nuchal cord.

A 5‐min Apgar score below 7 occurred in only one neonate in each group. The corresponding risk estimate had a very wide CI, indicating that the study could not reliably determine whether nuchal cord was associated with this uncommon outcome. Hoh et al. [16] also found that the presence of a nuchal cord did not necessarily translate into sustained neonatal compromise. The rarity of a persistently low Apgar score is compatible with evidence that compromise associated with an isolated nuchal cord may be transient and resolve after delivery and initial resuscitation [1, 2].

No intrapartum stillbirths occurred after enrollment. However, women presenting with confirmed intrauterine fetal death were excluded, and the study was not powered to assess this rare outcome. The absence of events therefore precluded estimation of a risk ratio and should not be interpreted as evidence that nuchal cord is unrelated to stillbirth. A systematic review found no clear association between nuchal cord overall and stillbirth, although risk may differ according to the number and tightness of cord loops [20]. Furthermore, nuchal cord alone is insufficient to attribute a fetal death to umbilical‐cord compromise without corroborating pathological evidence of vascular obstruction or fetal hypoxia [21]. Accordingly, this study cannot determine the relationship between nuchal cord and stillbirth.

Caesarean delivery was more frequent among women whose neonates had a nuchal cord, whereas spontaneous vaginal delivery was less frequent. This finding requires cautious interpretation because mode of delivery was not assessed using an adjusted model and the specific indications for caesarean delivery were not compared between groups. The difference could reflect fetal‐heart‐rate abnormalities, labor characteristics, clinician decision‐making or other obstetric indications. Previous studies have not consistently demonstrated that an isolated nuchal cord increases the need for caesarean delivery or justifies pre‐emptive operative birth [4, 7, 13]. Ultrasound detection of a nuchal cord should therefore prompt appropriate counseling, intrapartum surveillance and delivery‐room preparedness but should not, by itself, constitute an indication for induction, operative vaginal delivery or caesarean section.

Antenatal identification of a nuchal cord can cause considerable maternal anxiety because women may fear fetal strangulation or death. This anxiety may also influence clinicians' decisions regarding induction or operative delivery despite the generally favorable outcomes associated with an isolated nuchal cord [7, 8]. Clear, evidence‐based counseling is therefore essential to explain that ultrasound cannot predict whether an identified nuchal cord will tighten or cause fetal compromise.

Overall, the findings suggest that combined two‐dimensional and color Doppler ultrasound may be a useful adjunct for detecting nuchal cord among women assessed in early labor in a resource‐constrained referral setting. Its clinical value lies primarily in supporting counseling, intrapartum preparedness and timely neonatal support rather than predicting severe neonatal compromise or directing delivery solely on the basis of cord location. Larger multicentre studies using consecutive recruitment independent of ultrasound findings, adjusted analyses, cord‐blood gas measurements and neonatal follow‐up are needed to determine which nuchal‐cord characteristics identify neonates at greatest risk.

### 4.1 Study Strengths and Limitations

This study provides prospective evidence on the performance of two‐dimensional and color Doppler ultrasound for detecting nuchal cord in a resource‐limited Ugandan setting. Participants underwent a standardized ultrasound protocol and were followed through delivery, allowing comparison of the index test with direct examination of the umbilical cord as the reference standard. Complete diagnostic and immediate neonatal‐outcome data were available for all 246 participants, and the diagnostic measures were reported with 95% CIs to indicate their precision.

Several limitations should be considered. This was a single‐center study involving women with singleton term pregnancies presenting in early labor at a regional referral hospital. The findings may therefore not be generalizable to preterm or multiple pregnancies, women outside labor, other Ugandan hospitals or community settings. Umbilical arterial blood gases were not measured; consequently, neonatal hypoxia and metabolic acidosis could not be assessed objectively. Apgar scores and neonatal resuscitation were used as indicators of neonatal adaptation but are not specific measures of intrapartum hypoxia.

The neonatal‐outcome comparisons were secondary exploratory analyses based on unadjusted risk ratios. Differences in primigravidity, mode of delivery, number or tightness of cord loops and other maternal or intrapartum factors may have influenced the observed outcomes. Residual confounding therefore cannot be excluded, and the associations should not be interpreted as causal. Follow‐up was limited to the immediate post‐delivery period, precluding assessment of subsequent neonatal morbidity and longer‐term neurodevelopmental outcomes. Women presenting with confirmed intrauterine fetal death were excluded, and no intrapartum stillbirths occurred after enrollment; therefore, the study cannot determine whether nuchal cord is associated with stillbirth.

Recruitment followed a predetermined 1:2 ratio based on the ultrasound result rather than consecutive enrollment independent of the index test. Because sampling fractions from the complete screened population were unavailable, weighted estimates could not be calculated. This sampling approach may have influenced the observed frequency of nuchal cord and the unweighted estimates of sensitivity, specificity, area under the receiver operating characteristic curve and overall accuracy. The findings therefore require validation in a fully consecutive cohort recruited independently of the index‐test result. Finally, a separate research‐specific calibration or equipment‐performance assessment was not documented, although the same ultrasound machine and standardized preset settings were used throughout the study.

## 5 Conclusions and Recommendations

Two‐dimensional ultrasound combined with color Doppler demonstrated high diagnostic performance for detecting nuchal cord among women with singleton term pregnancies in early labor. Nuchal cord at delivery was associated with higher unadjusted risks of the composite immediate adverse neonatal outcome, particularly neonatal resuscitation and neonatal‐unit admission. However, these secondary exploratory findings may have been influenced by differences in mode of delivery and other unmeasured maternal or intrapartum factors and should not be interpreted as causal. Detection of a nuchal cord should be interpreted alongside fetal heart‐rate monitoring, labor progress and the overall clinical assessment. The finding may support delivery‐room preparedness and timely neonatal care but should not, in isolation, constitute an indication for operative delivery.

Larger multicentre prospective studies should determine whether ultrasound‐guided management improves maternal and neonatal outcomes. Future studies should distinguish single from multiple and loose from tight cord loops, adjust for relevant confounders, incorporate umbilical‐cord blood‐gas measurements and extend neonatal follow‐up beyond the immediate post‐delivery period.

## Author Contributions

**Fiona Asiimwe:** conceptualization, methodology, investigation, data curation and writing original draft. **Theoneste Hakizimana:** supervision, validation, interpretation and writing review and editing. **Marie Pascaline Sabine Ishimwe:** methodology, interpretation and writing review and editing. **Kato Paul Kalyebara, Leevan Tibaijuka, Joseph Ngonzi, Henry Lugobe, Godfrey Twesigomwe, Kahimakazi Irene, Jjuuko Mark, Musa Kayondo** and **Julius Mugisha:** investigation, data curation and writing review and editing. **Rogers Kajabwangu:** supervision, validation and writing review and editing. All authors read and approved the final manuscript.

## Funding

The authors have nothing to report.

## Disclosure

All authors have read and approved the final version of the manuscript. Theoneste Hakizimana, the corresponding author and manuscript guarantor, had full access to all the data in this study and takes complete responsibility for the integrity of the data and the accuracy of the data analysis.

## Ethics Statement

The research was conducted in accordance with the ethical principles outlined in the Declaration of Helsinki.

## Consent

Written informed consent was obtained from all participants prior to enrollment. The research was conducted in accordance with the ethical principles outlined in the Declaration of Helsinki. Confidentiality and anonymity were strictly upheld throughout the study.

## Conflicts of Interest

The authors declare no conflicts of interest.

## Transparency Statement

Theoneste Hakizimana, the manuscript guarantor, affirms that this manuscript is an honest, accurate and transparent account of the study being reported; that no important aspects of the study have been omitted; and that any discrepancies from the study as planned have been explained.

## Data Availability

Datasets are available from the corresponding author on reasonable request. Dr. Theoneste Hakizimana (email: theonestehakizimana5@gmail.com). The datasets supporting the findings of this study are available from the corresponding author, Dr. Theoneste Hakizimana, upon reasonable request. (email: theonestehakizimana5@gmail.com).
